# Supplementary material for: Efficient polynomial analysis of magic-angle spinning sidebands and application to order parameter determination in anisotropic samples
Source: Magn Reson (Gott). 2021 Aug 4;2(2):589–606. doi: 10.5194/mr-2-589-2021 (PMC10539843; doi:10.5194/mr-2-589-2021)
Supplement: The supplement related to this article is available online at: https://doi.org/10.5194/mr-2-589-2021-supplement. [file mr-2-589-supplement.pdf]

Supplement of Magn. Reson., 2, 589–606, 2021
https://doi.org/10.5194/mr-2-589-2021-supplement
© Author(s) 2021. CC BY 4.0 License.

Open Access
MAGNETIC RESONANCE

Supplement of

Efficient polynomial analysis of magic-angle spinning sidebands and application to order parameter determination in anisotropic samples

Günter Hempel et al.

Correspondence to: Günter Hempel (guenter.hempel@physik.uni-halle.de)

The copyright of individual parts of the supplement might differ from the article licence.

S1. AVERAGES OVER POWER PRODUCTS OF TRIGONOMETRIC FUNCTIONS

A. Azimuthal average

1. *Proposition*

$$\left\langle \sin^m\!\gamma \, \cos^n\!\gamma \right\rangle_\gamma = \begin{cases} \dfrac{(m-1)!!\,(n-1)!!}{(m+n)!!} & \text{if } m \text{ and } n \text{ even} \\[2ex] 0 & \text{otherwise} \end{cases} \tag{1}$$

Meaning of the averaging symbol:

$$\left\langle A\left(\gamma\right)\right\rangle_\gamma = \frac{1}{2\pi} \int\limits_a^{a+2\pi} A\left(\gamma\right)\ \mathrm{d}\gamma \quad ; \quad a \in \mathbb{R} \tag{2}$$

For sake of shortness of the expressions, the index γ is omitted in the following.

2. *Proof for odd exponents:*

If the sine power m is odd:
We set a = - π, split the integration range in [- π , 0] and [0 , π] and substitute in the first part $\gamma \to$ - γ:

$$\int\limits_{-\pi}^{0} \sin^m\!\gamma \, \cos^n\!\gamma \ \mathrm{d}\gamma + \int\limits_{0}^{\pi} \sin^m\!\gamma \, \cos^n\!\gamma \ \mathrm{d}\gamma = \int\limits_{\pi}^{0} \sin^m(-\gamma) \, \cos^n(-\gamma) \ \mathrm{d}(-\gamma) + \int\limits_{0}^{\pi} \sin^m\!\gamma \, \cos^n\!\gamma \ \mathrm{d}\gamma = 0 \tag{3}$$

If the cosine power n is odd: We substitute $\gamma \to \pi/2 - \gamma$ and get again an integral with odd sine power which therefore will be zero likewise.

3. *Proof for even exponents*

This will be done by mathematical induction from n to $n+2$ with base case $n = 0$. The latter has to be proven with an induction from m to $m+2$.
Base case $n = 0$: The proposition eqn. (1) has here the form:

$$\left\langle \sin^m\!\gamma \right\rangle = \frac{(m-1)!!}{m\ !!} \tag{4}$$

To prove this by a further induction we check first that the base case is obviously valid for $m = 0$. The inductive step $m \to m+2$ is done by

$$\begin{aligned} \textstyle\int \sin^{m+2}\!\gamma \ \mathrm{d}\gamma &= -\cos\gamma \, \sin^{m+1}\!\gamma + (m+1) \ \textstyle\int \cos^2\!\gamma \, \sin^m\!\gamma \ \mathrm{d}\gamma \\ &= -\cos\gamma \, \sin^{m+1}\!\gamma + (m+1) \ \textstyle\int \sin^m\!\gamma \ \mathrm{d}\gamma - (m+1) \ \textstyle\int \sin^{m+2}\!\gamma \ \mathrm{d}\gamma \end{aligned} \tag{5}$$

Inserting the integration limits: first term at the right-hand side is cancelled, and we get

$$\left\langle \sin^{m+2}\!\gamma \right\rangle = \frac{m+1}{m+2} \left\langle \sin^m\!\gamma \right\rangle = \frac{m+1}{m+2} \ \cdot \ \frac{(m-1)!!}{m\ !!} = \frac{(m+1)!!}{(m+2)!!} \tag{6}$$

That means, the validity of the sub-proposition (4) for m implies the validity of that also for $m+2$. This proves sub-proposition (4) for all even m. Therefore the base case for the following induction is valid.
Inductive step $n \to n+2$: Suppose proposition (1) is valid for a particular n. We investigate this expression for $n \to n+2$ by replacing $\cos^{n+2}\!\gamma = \cos^n\!\gamma \left(1 - \sin^2\!\gamma\right)$:

$$\left\langle \sin^m\gamma \cos^{n+2}\gamma \right\rangle = \left\langle \sin^m\gamma \cos^n\gamma \right\rangle - \left\langle \sin^{m+2}\gamma \cos^n\gamma \right\rangle = \frac{(m-1)!!\,(n-1)!!}{(m+n)!!} - \frac{(m+1)!!\,(n-1)!!}{(m+2+n)!!}$$
$$= \frac{(m-1)!!\,(n-1)!!}{(m+2+n)!!}\left[(m+n+2)-(m+1)\right] = \frac{(m-1)!!\,(n+1)!!}{(m+2+n)!!} \tag{7}$$

which is exactly the proposition for $n+2$.
The validity of proposition (1) for n implies the validity of the proposition also for $n+2$. This proves that proposition for all even m and n.

□

B. Polar average

1. *Proposition*

$$\left\langle \cos^n\alpha \sin^m\alpha \right\rangle_{\cos\alpha} = \begin{cases} \dfrac{m!!\,(n-1)!!}{(m+n+1)!!} & \text{if } m \text{ and } n \text{ even} \\ \dfrac{m!!\,(n-1)!!}{(m+n+1)!!}\cdot\dfrac{\pi}{2} & \text{if } m \text{ odd and } n \text{ even} \\ 0 & \text{if } n \text{ odd} \end{cases} \tag{8}$$

Meaning of the averaging symbol:

$$\left\langle A(\alpha) \right\rangle_{\cos\alpha} = \frac{1}{\pi}\int_0^\pi A(\alpha)\,\sin\alpha\,\mathrm{d}\alpha = \frac{1}{2}\int_{-1}^{1} A(\alpha)\,\mathrm{d}(\cos\alpha) \tag{9}$$

For sake of shortness of the expressions, the index $\cos\alpha$ is omitted in the following.

For odd n: Split of the integration interval into the parts $[0,\pi/2]$ and $[\pi/2,\pi]$; in the second part: substitution $\alpha\to\pi-\alpha$, then $\mathrm{d}\alpha$ and $\cos\alpha$ alternate the sign:

$$\left\langle \cos^n\alpha\ \sin^m\alpha \right\rangle = \frac{1}{\pi}\int_0^\pi \cos^n\alpha\ \sin^{m+1}\alpha\,\mathrm{d}\alpha = \frac{1}{\pi}\int_0^{\pi/2} \cos^{\mathrm{n}}\alpha\ \sin^{\mathrm{m}+1}\alpha\,\mathrm{d}\alpha + \frac{1}{\pi}\int_{\pi/2}^{\pi} \cos^{\mathrm{n}}\alpha\,\sin^{\mathrm{m}+1}\alpha\,\mathrm{d}\alpha$$
$$= \frac{1}{\pi}\int_0^{\pi/2} \cos^n\alpha\ \sin^{m+1}\alpha\,\mathrm{d}\alpha + \frac{1}{\pi}\int_{\pi/2}^{0} \cos^{\mathrm{n}}\alpha\ \sin^{\mathrm{m}+1}\alpha\,\mathrm{d}\alpha = 0 \tag{10}$$

This proves proposition (8) for odd n.

For even n: Prove by mathematical induction $m\to m+2$; therefore two base cases (here $m=0$ and $m=1$) are needed.

Base case 1 ($m=0$): Substitution $\cos\alpha\to c$:

$$\left\langle \cos^n\alpha \right\rangle = \frac{1}{2}\int_{-1}^{1} c^n\,\mathrm{d}c = \frac{1}{2}\left[\frac{1}{n+1}c^{n+1}\right]_{-1}^{1} = \frac{1}{n+1} \tag{11}$$

which proves proposition (8) for $m=0$ and $n\in\mathbb{N}$.

Base case 2 ($m=1$): To be shown here:

$$\left\langle \cos^n\alpha \sin\alpha \right\rangle = \frac{(n-1)!!}{(n+2)!!}\cdot\frac{\pi}{2} \tag{12}$$

This is done by the following steps:

$$\left\langle \cos^{n+2}\alpha \ \sin\alpha \right\rangle = \frac{1}{2} \int_0^\pi \cos^{n+2}\alpha \sin^2\alpha \ \mathrm{d}\alpha = \frac{1}{2} \int_0^\pi \left(\cos^{n+2}\alpha - \cos^{n+4}\alpha \right) \ \mathrm{d}\alpha \tag{13}$$

Partial integration of a cosine power:

$$\int_0^\pi \cos^p\alpha \ \mathrm{d}\alpha = \left[\sin\alpha \cos^{p-1}\alpha \right]_0^\pi - (p-1) \int_0^\pi \left(-\sin^2\alpha \right) \ \cos^{p-2}\alpha \ \mathrm{d}\alpha = (p-1) \ \cdot \ 2 \left\langle \cos^{p-2}\alpha \ \sin\alpha \right\rangle \tag{14}$$

Inserting this rule into eqn. (13):

$$\left\langle \cos^{p+2}\alpha \ \sin\alpha \right\rangle = (p+1) \left\langle \cos^p\alpha \ \sin\alpha \right\rangle - (p+3) \left\langle \cos^{p+2}\alpha \ \sin\alpha \right\rangle \tag{15}$$

or

$$\left\langle \cos^{n+2}\alpha \ \sin\alpha \right\rangle = \frac{n+1}{n+4} \left\langle \cos^n\alpha \ \sin\alpha \right\rangle = \frac{n+1}{n+4} \cdot \frac{(n-1)!!}{(n+2)!!} \cdot \frac{\pi}{2} = \frac{(n+1)!!}{(n+4)!!} \cdot \frac{\pi}{2} \tag{16}$$

It can be shown easily that eqn. (12) is fulfilled for $n = 0$. This together with eqn. (16) as induction step proves base case 2.

Inductive step $m \to m+2$: Suppose proposition (8) would be valid for a particular m and all even n. We calculate the average for $m+2$ by replacing $\sin^{m+2}\gamma = \sin^m\gamma \left(1 - \cos^2\gamma\right)$:

$$\begin{aligned} \left\langle \cos^n\alpha \sin^{m+2}\alpha \right\rangle &= \left\langle \cos^n\alpha \sin^m\alpha \right\rangle - \left\langle \cos^{n+2}\alpha \sin^m\alpha \right\rangle \\ &= \frac{m!! \, (n-1)!!}{(m+n+1)!!} \ f_n - \frac{m!! \, (n+1)!!}{(m+n+3)!!} \ f_n \\ &= \frac{m!! \, (n-1)!!}{(m+n+3)!!} \left[(m+n+3) - (n+1) \right] \ f_n \\ &= \frac{(m+2)!! \, (n-1)!!}{(m+n+3)!!} \ f_n \end{aligned} \tag{17}$$

($f_n := 1$ for even n and $\pi/2$ for odd n) which is exactly proposition (8) for $m \to m+2$.

This inductive step together with the proven base cases proves proposition (8) $\forall \{m, n\} \subset \mathbb{N}$.

□

S2. POWDER-AVERAGED PHASE POWERS

Listed up to eigth power:

$$\langle \Phi \rangle = 0 \tag{18}$$

$$\left\langle \Phi^2 \right\rangle = \left(\frac{\delta\omega_0}{\omega_r}\right)^2 \cdot \frac{1}{45}\left(3+\eta^2\right)\left(5+\cos\gamma_r\right)\left(1-\cos\gamma_r\right) \tag{19}$$

$$\left\langle \Phi^3 \right\rangle = \left(\frac{\delta\omega_0}{\omega_r}\right)^3 \cdot \frac{4}{5\cdot 7}\left(1-\eta^2\right)\sin\gamma_r \sin^2\frac{\gamma_r}{2} \tag{20}$$

$$\left\langle \Phi^4 \right\rangle = \left(\frac{\delta\omega_0}{\omega_r}\right)^4 \cdot \frac{4}{945}\left(3+\eta^2\right)^2\left(5+\cos\gamma_r\right)^2 \sin^4\frac{\gamma_r}{2} \tag{21}$$

$$\left\langle \Phi^5 \right\rangle = \left(\frac{\delta\omega_0}{\omega_r}\right)^5 \cdot \frac{32}{693}\left(3+\eta^2\right)\left(1-\eta^2\right)\left(5+\cos\gamma_r\right)\sin\gamma_r \sin^4\frac{\gamma_r}{2} \tag{22}$$

$$\begin{aligned}\left\langle \Phi^6 \right\rangle = &\left(\frac{\delta\omega_0}{\omega_r}\right)^6 \cdot \left[\frac{1}{16016}\left(5515+\frac{16381}{3}\eta^2+\frac{49963}{27}\eta^4+\frac{49471}{243}\eta^6\right)\right.\\ &-\frac{1}{286}\left(131+\frac{1009}{7}\eta^2+\frac{2567}{63}\eta^4+\frac{2843}{567}\eta^6\right)\cos\gamma_r+\frac{1}{32032}\left(2983+6219\eta^2+\frac{2477}{9}\eta^4+\frac{12185}{81}\eta^6\right)\cos 2\gamma_r\\ &+\frac{1}{12012}\left(397-\frac{1237}{3}\eta^2+\frac{8429}{27}\eta^4+\frac{1145}{243}\eta^6\right)\cos 3\gamma_r-\frac{1}{16016}\left(179-\frac{235}{3}\eta^2+\frac{3155}{27}\eta^4+\frac{839}{243}\eta^6\right)\cos 4\gamma_r\\ &\left.-\frac{1}{143}\left(\frac{5}{28}+\frac{19}{84}\eta^2+\frac{37}{756}\eta^4+\frac{7}{972}\eta^6\right)\cos 5\gamma_r-\frac{1}{24}\frac{1}{143}\left(\frac{5}{28}+\frac{19}{84}\eta^2+\frac{37}{756}\eta^4+\frac{7}{972}\eta^6\right)\cos 6\gamma_r\right]\end{aligned} \tag{23}$$

$$\left\langle \Phi^7 \right\rangle = \left(\frac{\delta\omega_0}{\omega_r}\right)^7 \cdot \frac{64}{3861}\left(\eta^2-1\right)\left(3+\eta^2\right)^2\left(5+\cos\gamma_r\right)^2\cos\frac{\gamma_r}{2}\sin^7\frac{\gamma_r}{2} \tag{24}$$

$$\begin{aligned}\left\langle \Phi^8 \right\rangle = &\left(\frac{\delta\omega_0}{\omega_r}\right)^8 \cdot \frac{1}{15949791}\cdot 4\left(3+\eta^2\right)\left(5+\cos\gamma_r\right)\left[1274778+282042\eta^2+645534\eta^4+34958\eta^6\right.\\ &+3\left(296217-132759\eta^2+194067\eta^4+5675\eta^6\right)\cos\gamma_r+51030\cos 2\gamma_r+1701\cos 3\gamma_r\\ &\left.+\eta^2\left(2997+279\eta^2+79\eta^4\right)\left(30\cos 2\gamma_r+\cos 3\gamma_r\right)\right]\sin^8\frac{\gamma_r}{2}\end{aligned} \tag{25}$$

S3. MATHEMATICA EXPRESSIONS FOR EVALUATION OF THE GENERAL SSB POLYNOMIAL

The following passages can be copied and pasted into Mathematica, possibly with an in-between step of pasting the text into a simple text editor to remove formatting information. Note that on some systems it seems that the curly brackets { and } are pasted as letters f and g, respectively. Since f and g do not appear in the formulae, search-and-replace can be used.

First, we define an auxiliary function with the independent variables #1, #2 and #3 being identified with n, k, and η, respectively:

```
aux = Sum[#2!/(p!q!(#2 - p - q)!)*Binomial[#1 - 2*#2, r]2^(4*#2 + r + p)/6^#1*(-1)^r*(p + r - 1)!!(q + #1 - 2*#2 - r - 1)!!/(p + q + #1 - 2*#2)!!(1 + (-1)^(r - p))(1 + (-1)^(q + #1 + 2*#2 - r))/4*Sum[(-1)^(q - s + u)*Binomial[q,s]*Binomial[#2 - p - q, t]*Binomial[#1 - 2*#2 - r, u]*(2*#2)!! (p + r + 2*s + 2*t + 2*u - 1)!!/(2*#2 + p + r + 2*s + 2*t + 2*u + 1)!!*Sum[(-1)^v*3^(#1 - 2*#2 + p - r + 2*s + 2*t - v - w)*Binomial[p + 2*s + 2*t + u, v] Binomial[#1 - 2*#2 - r - u, w]*#3^(v + w + 2*#2 - p - 2*s - 2*t + r)*(v + w - 1)!! (2*#2 - p - 2*s - 2*t + r - 1)!!/(v + w + 2*#2 - p - 2*s - 2*t + r)!!*(1 + (-1)^(v + w))*(1 + (-1)^(r - p))/4, {w, 0, #1 - 2*#2 - r - u}, {v, 0, p + 2*s + 2*t + u}],{u,0,#1-2*#2-r},{t,0,#2-p-q},{s,0,q}],{r,0,#1-2*#2},{q,0,#2},{p,0,#2}]&;
```

The general function for SSB generation, with #1, #2, #3 and #4 identified with the SSB order m, $\delta\omega_0/\omega_\mathrm{r}$, η and the maximum order n of the polynomial, respectively, is then:

```
rsb = Sum[1/2^n*#2^n*(-1)^(n - k - #1 - b)/((n - k - #1 - 2*b)!*(3*k - n + #1 + 2b)!(n - 2*k - b)!*b!)*aux[n, k, #3], {n, #1, #4}, {k, 0, n/2}, {b, 0, n - 2*k}] &;
```

With this, one can readily generate the SSB polynomials,
e.g. for the centerband ($m = 0$) up to order $n = 6$:

```
rsb[0, delta, eta, 6] // Simplify
```

S4. SPINNING SIDEBAND INTENSITIES

Equations for SSB intensities using polynomials up to 12th order in $\omega_0/\omega_\mathrm{r}$ (abbreviations: $K_1 := 3+\eta^2$, $K_2 := 1-\eta^2$ and $w = \delta\omega_0/\omega_\mathrm{r}$).

$$\begin{aligned} I_0 = 1 &- \frac{K_1^2}{20}w^2 + \frac{227\ K_1^2}{181\ 440}w^4 - \frac{49\ 471\ K_1^3 + 4\ 428\ K_2^2}{2\ 802\ 159\ 360}w^6 + \frac{K_1\left(1\ 466\ 405\ K_1^3 - 709\ 776\ K_2^2\right)}{9\ 146\ 248\ 151\ 040}w^8 \\ &- \frac{K_1^2\left(286\ 311\ 167\ K_1^3 - 494\ 915\ 400\ K_2^2\right)}{281\ 521\ 518\ 089\ 011\ 200}w^{10} \\ &+ \frac{998\ 271\ 153\ 509\ K_1^6 - 2\ 160\ K_2^2\left(1\ 577\ 931\ 893\ K_1^3 + 218\ 222\ 883\ K_2^2\right)}{209\ 789\ 835\ 279\ 931\ 146\ 240\ 000}w^{12} \end{aligned} \tag{26}$$

$$\begin{aligned} I_{\pm 1} =& \frac{K_1}{45}w^2 \mp \frac{K_2}{105}w^3 - \frac{17\ K_1^2}{22\ 680}w^4 \pm \frac{23\ K_1K_2}{83\ 160}w^5 + \frac{2\ 843\ K_1^3 - 2\ 484\ K_2^2}{233\ 513\ 280}w^6 \\ &\mp \frac{19\ K_1^2K_2}{5\ 189\ 184}w^7 - \frac{K_1\left(123\ 823\ K_1^3 - 285\ 552K_2^2\right)}{1\ 028\ 952\ 916\ 992}w^8 \pm \frac{K_2\left(959\ 357\ K_1^3 - 196\ 884\ K_2^2\right)}{32\ 583\ 509\ 038\ 080}w^9 \\ &+ \frac{K_1^2\left(11\ 362\ 895\ K_1^3 - 46\ 559\ 016\ K_2^2\right)}{14\ 076\ 075\ 904\ 450\ 560}w^{10} \mp \frac{K_1K_2\left(766\ 057\ K_1^3 - 663\ 984\ K_2^2\right)}{4\ 692\ 025\ 301\ 483\ 520}w^{11} \\ &- \frac{34\ 324\ 127\ 551\ K_1^6 - 2\ 160\ K_2^2\left(96\ 357\ 427\ K_1^3 + 2\ 033\ 937\ K_2^2\right)}{8\ 741\ 243\ 136\ 663\ 797\ 760\ 000}w^{12} \end{aligned} \tag{27}$$

$$\begin{aligned} I_{\pm 2} =& \frac{K_1}{360}w^2 \pm \frac{K_2}{210}w^3 + \frac{K_1^2}{12\ 960}w^4 \mp \frac{17\ K_1K_2}{83\ 160}w^5 - \frac{12185\ K_1^3 - 87372\ K_2^2}{3736212480}w^6 \\ &\pm \frac{49\ K_1^2K_2}{14826240}w^7 + \frac{(3+\eta^2)\left(966239\ K_1^3 - 10569744K_2^2\right)}{20579058339840}w^8 \mp \frac{K_2\left(990181\ K_1^3 - 583956\ K_2^2\right)}{32\ 583\ 509\ 038\ 080}w^9 \\ &- \frac{(3+\eta^2)^2\left(14591857\ K_1^3 - 197993592\ K_2^2\right)}{37536202411868160}w^{10} \pm \frac{K_1K_2\left(4464013\ K_1^3 - 7892208\ K_2^2\right)}{23981462652026880}w^{11} \\ &+ \frac{75617921797\ K_1^6 - 2\ 160\ K_2^2\left(548505769\ (3+\eta^2)^3 - 72353061\ K_2^2\right)}{349649725466551910400000}w^{12} \end{aligned} \tag{28}$$

$$\begin{aligned} I_{\pm 3} =& \frac{K_1^2}{22\ 680}w^4 \pm \frac{K_1K_2}{27720}w^5 - \frac{1\ 145\ K_1^3 + 65556\ K_2^2}{4203239040}w^6 \mp \frac{163\ K_1^2K_2}{155675520}w^7 \\ &- \frac{K_1\left(9379\ K_1^3 - 682128K_2^2\right)}{1714921528320}w^8 \pm \frac{K_2\left(2845\ K_1^3 - 5076\ K_2^2\right)}{212964111360}w^9 \\ &+ \frac{K_1^2\left(224699\ K_1^3 - 10360440\ K_2^2\right)}{2346012650741760}w^{10} \mp \frac{K_1K_2\left(1832201\ K_1^3 - 7529328\ K_2^2\right)}{17986096989020160}w^{11} \\ &- \frac{19307643899\ K_1^6 - 2\ 160\ K_2^2\left(360943523\ K_1^3 - 121172787\ K_2^2\right)}{262237294099913932800000}w^{12} \end{aligned} \tag{29}$$

$$\begin{aligned} I_{\pm 4} =& \frac{K_1^2}{362880}w^4 \pm \frac{K_1K_2}{166320}w^5 + \frac{839\ K_1^3 + 20844\ K_2^2}{5604318720}w^6 \mp \frac{K_1^2K_2}{77837760}w^7 \\ &- \frac{K_1\left(58999\ K_1^3 + 5681232K_2^2\right)}{41158116679680}w^8 \pm \frac{K_2\left(2845\ K_1^3 - 5076\ K_2^2\right)}{32583509038080}w^9 \\ &+ \frac{K_1^2\left(34081\ K_1^3 - 18031032\ K_2^2\right)}{9384050602967040}w^{10} \pm \frac{K_1K_2\left(233311\ K_1^3 - 2825712\ K_2^2\right)}{8993048494510080}w^{11} \\ &+ \frac{11419571509\ K_1^6 - 2\ 160\ K_2^2\left(656644693\ K_1^3 - 496531917\ K_2^2\right)}{932399267910805094400000}w^{12} \end{aligned} \tag{30}$$

S5. 2D OSCILLATION COEFFICIENTS

A. Belonging to I_0

$$C_{00} = I_0^{(\mathrm{iso})} + \left(\frac{\omega_0\delta}{\omega_r}\right)^2 \left(\frac{1}{12}\, Q_2{}^{(2)} \langle P_2\rangle - \frac{31}{96}\, Q_4{}^{(2)} \langle P_4\rangle\right) + \left(\frac{\omega_0\delta}{\omega_r}\right)^4 \left(\frac{85}{528}\, Q_{11} \langle P_2\rangle + Q_{21} \langle P_4\rangle + \frac{59}{3\,024}\, Q_3 \langle P_6\rangle + \frac{19\,733}{7\,248\,384}\, Q_4 \langle P_8\rangle\right) \tag{31}$$

$$C_{02} = \left(\frac{\omega_0\delta}{\omega_r}\right)^2 \frac{3\, Q_2^{(2)} \langle P_2\rangle + 5\, Q_4^{(2)} \langle P_4\rangle}{18} + \left(\frac{\omega_0\delta}{\omega_r}\right)^4 \cdot \frac{1}{12}\left(Q_{12} \langle P_2\rangle - Q_{22} \langle P_4\rangle + \frac{1}{2}\, Q_3 \langle P_6\rangle - \frac{1}{11}\, Q_4 \langle P_8\rangle\right) \tag{32}$$

$$C_{04} = -\left(\frac{\omega_0\delta}{\omega_r}\right)^2 \frac{35}{576}\, Q_4^{(2)} \langle P_4\rangle + \left(\frac{\omega_0\delta}{\omega_r}\right)^4 \cdot \frac{1}{12}\left(Q_{23} \langle P_4\rangle + \frac{1}{15}\, Q_3 \langle P_6\rangle + \frac{111}{260}\, Q_4 \langle P_8\rangle\right) \tag{33}$$

$$C_{06} = \left(\frac{\omega_0\delta}{\omega_r}\right)^4 \cdot \frac{11\, Q_3 \langle P_6\rangle + 6\, Q_4 \langle P_8\rangle}{360} \tag{34}$$

$$C_{08} = \left(\frac{\omega_0\delta}{\omega_r}\right)^4 \cdot \frac{Q_4\ \langle P_8\rangle}{768} \tag{35}$$

$$S_{02} = -i\, \frac{\omega_0\delta}{\omega_r}\, \frac{1}{2\sqrt{6}} E_5 \langle P_2\rangle + i\, \left(\frac{\omega_0\delta}{\omega_r}\right)^3 \frac{3\,344\sqrt{3}\ \left(3+\eta^2\right) \langle P_2\rangle\ E_5\ - 296\ \langle P_4\rangle\, Q_4^{(3)} + 875\ \langle P_6\rangle\, Q_6^{(3)}}{532\,224\sqrt{2}} \tag{36}$$

$$S_{0;4} = -i \left(\frac{\omega_0\delta}{\omega_r}\right)^3 \frac{\langle P_4\rangle\, Q_4^{(3)} + 6\ \langle P_6\rangle\, Q_6^{(3)}}{4\,752\sqrt{2}} \tag{37}$$

$$S_{0;6} = i \left(\frac{\omega_0\delta}{\omega_r}\right)^3 \frac{Q_6^{(3)} \langle P_6\rangle}{6\,912\sqrt{2}} \tag{38}$$

$$S_{08} = 0 \tag{39}$$

B. Belonging to $I_{\pm 1}$

$$C_{\pm 10} = I_{\pm 1}^{(\mathrm{iso})} + \left(\frac{\omega_0\delta}{\omega_r}\right)^2 \cdot \frac{1}{18}\left[-\, Q_2^{(2)} \langle P_2\rangle + 3\, Q_4{}^{(2)} \langle P_4\rangle\right] \pm \left(\frac{\omega_0\delta}{\omega_r}\right)^3\ \frac{21\, Q_4^{(3)} \langle P_4\rangle - 50\, Q_6^{(3)} \langle P_6\rangle}{83\,160\sqrt{2}} - \left(\frac{\omega_0\delta}{\omega_r}\right)^4 \left[\frac{31}{264}\, Q_{11} \langle P_2\rangle + Q_{24} \langle P_4\rangle - \frac{47}{1\,512}\, Q_3 \langle P_6\rangle + \frac{434}{10296}\, Q_4 \langle P_8\rangle\right] \tag{40}$$

$$C_{\pm 1;2} = -\left(\frac{\omega_0\delta}{\omega_r}\right)^2 \cdot \frac{3\, Q_2^{(2)} \langle P_2\rangle +\ Q_4^{(2)} \langle P_4\rangle}{18} \pm \left(\frac{\omega_0\delta}{\omega_r}\right)^3 \frac{44\sqrt{3}\,(3+\eta^2)\ \langle P_2\rangle\ E_5 - 5\,\langle P_4\rangle\, Q_4^{(3)} + 14\,\langle P_6\rangle\, Q_6^{(3)}}{33\,264\sqrt{2}} + \left(\frac{\omega_0\delta}{\omega_r}\right)^4 \frac{(Q_{14} -\ Q_{13})\,\langle P_2\rangle - (Q_{25} + Q_{26})\,\langle P_4\rangle + \frac{3}{2} Q_3 \langle P_6\rangle + \frac{215}{3\,423}\, Q_4 \langle P_8\rangle}{24} \tag{41}$$

$$C_{\pm 1;4} = \pm \left(\frac{\omega_0\delta}{\omega_r}\right)^3 \frac{\langle P_4\rangle\, Q_4^{(3)} + 6\,\langle P_6\rangle\, Q_6^{(3)}}{4\ 752\sqrt{2}} + \left(\frac{\omega_0\delta}{\omega_r}\right)^4 \left[\frac{1}{24}\left(Q_{27}+Q_{28}\right)\langle P_4\rangle + \frac{41}{360}\, Q_3\,\langle P_6\rangle - \frac{4}{195}\, Q_4\,\langle P_8\rangle\right] \tag{42}$$

$$C_{\pm 1;6} = \left(\frac{\omega_0\delta}{\omega_r}\right)^4 \frac{1}{120}\left(\frac{11}{6}\, Q_3\,\langle P_6\rangle + \ Q_4\,\langle P_8\rangle\right) \tag{43}$$

$$C_{\pm 1;8} = 0 \tag{44}$$

$$\begin{aligned} S_{\pm 1;2} = &\mp i\left(\frac{\omega_0\delta}{\omega_r}\right)^2 \frac{3\ Q_2^{(2)}\,\langle P_2\rangle + \ Q_4^{(2)}\,\langle P_4\rangle}{18} - i\left(\frac{\omega_0\delta}{\omega_r}\right)^3 \frac{44\sqrt{3}\left(3+\eta^2\right)\langle P_2\rangle\ \ E_5\ \ - 5\,\langle P_4\rangle\, Q_4^{(3)} + 14\,\langle P_6\rangle\, Q_6^{(3)}}{33\ 264\sqrt{2}} \\ &\mp i\left(\frac{\omega_0\delta}{\omega_r}\right)^4 \frac{1}{24}\left[\left(Q_{14}+Q_{13}\right)\langle P_2\rangle + \left(Q_{25}-Q_{26}\right)\langle P_4\rangle - \frac{1}{2}Q_3\,\langle P_6\rangle - \frac{19}{489}\, Q_4\,\langle P_8\rangle\right] \end{aligned} \tag{45}$$

$$S_{\pm 1;4} = i\left(\frac{\omega_0\delta}{\omega_r}\right)^3 \frac{\langle P_4\rangle\, Q_4^{(3)} + 6\,\langle P_6\rangle\, Q_6^{(3)}}{4\ 752\sqrt{2}} \pm i\left(\frac{\omega_0\delta}{\omega_r}\right)^4 \left[\frac{1}{24}\left(Q_{27}-Q_{28}\right)\langle P_4\rangle + \frac{7}{72}\, Q_3\,\langle P_6\rangle - \frac{1}{60}\, Q_4\,\langle P_8\rangle\right] \tag{46}$$

$$S_{\pm 1;6} = \pm i\left(\frac{\omega_0\delta}{\omega_r}\right)^4 \frac{11\ Q_3\,\langle P_6\rangle + 6\ Q_4\,\langle P_8\rangle}{720} \tag{47}$$

$$S_{\pm 18} = 0 \tag{48}$$

C. Belonging to $I_{\pm 2}$

$$\begin{aligned} C_{\pm 20} = &I_2^{(\mathrm{iso})} + \left(\frac{\omega_0\delta}{\omega_r}\right)^2 \left[\frac{1}{72}\, {Q_2}^{(2)}\,\langle P_2\rangle - \frac{1}{192}\, {Q_4}^{(2)}\,\langle P_4\rangle\right] \mp \left(\frac{\omega_0\delta}{\omega_r}\right)^3 \frac{21\,\langle P_4\rangle\, Q_4^{(3)} - 50\,\langle P_6\rangle\, Q_6^{(3)}}{166\ 320\sqrt{2}} \\ &+ \left(\frac{\omega_0\delta}{\omega_r}\right)^4 \left[\frac{1}{24}Q_{11}\,\langle P_2\rangle + \frac{1}{24}Q_{29}\,\langle P_4\rangle - \frac{7}{216}\, Q_3\,\langle P_6\rangle + \frac{203}{14\ 976}\, Q_4\,\langle P_8\rangle\right] \end{aligned} \tag{49}$$

$$\begin{aligned} C_{\pm 2;2} = &\pm \frac{\omega_0\delta}{\omega_r}\ \frac{1}{4\sqrt{6}} E_5\,\langle P_2\rangle + \left(\frac{\omega_0\delta}{\omega_r}\right)^2 \frac{1}{36}\left(3\ {Q_2}^{(2)}\,\langle P_2\rangle + \ {Q_4}^{(2)}\,\langle P_4\rangle\right) \\ &\mp \left(\frac{\omega_0\delta}{\omega_r}\right)^3 \frac{1\ 584\sqrt{3}\ \left(3+\eta^2\right)\ E_5\ \ \langle P_2\rangle - 152\,\langle P_4\rangle\, Q_4^{(3)} + 441\,\langle P_6\rangle\, Q_6^{(3)}}{532\ 224\sqrt{2}} \\ &+ \left(\frac{\omega_0\delta}{\omega_r}\right)^4 \frac{1}{24}\left[\left(\frac{1}{2}\ Q_{12} + Q_{15}\right)\langle P_2\rangle + \left(Q_{22}-Q_{210}\right)\langle P_4\rangle - \frac{128}{143}\, Q_4\,\langle P_8\rangle\right] \end{aligned} \tag{50}$$

$$C_{\pm 2;4} = \left(\frac{\omega_0\delta}{\omega_r}\right)^2 \frac{1}{576}\, {Q_4}^{(2)}\,\langle P_4\rangle + \left(\frac{\omega_0\delta}{\omega_r}\right)^4 \frac{1}{24}\left[\left(\frac{1}{8}Q_{28} + Q_{211}\right)\langle P_4\rangle + \frac{521}{120}\, Q_3\,\langle P_6\rangle + \frac{1\ 159}{2\ 080}\, Q_4\,\langle P_8\rangle\right] \tag{51}$$

$$C_{\pm 2;6} = \mp \left(\frac{\omega_0\delta}{\omega_r}\right)^3 \frac{\langle P_6\rangle\, Q_6^{(3)}}{4608\sqrt{2}} + \left(\frac{\omega_0\delta}{\omega_r}\right)^4 \left[\frac{11}{1\ 440}\, Q_3\,\langle P_6\rangle + \frac{1}{240}\, Q_4\,\langle P_8\rangle\right] \tag{52}$$

$$C_{\pm 2;8} = -\frac{1}{768}\left(\frac{\omega_0\delta}{\omega_r}\right)^4\, Q_4\,\langle P_8\rangle \tag{53}$$

$$S_{\pm 2;2} = \; i\,\frac{\omega_0\delta}{\omega_r}\,\frac{1}{4\sqrt{6}}E_5\left\langle P_2\right\rangle \pm \frac{i}{36}\left(\frac{\omega_0\delta}{\omega_r}\right)^2\left(3\,{Q_2}^{(2)}\left\langle P_2\right\rangle + \;{Q_4}^{(2)}\left\langle P_4\right\rangle\right)$$
$$-\,i\left(\frac{\omega_0\delta}{\omega_r}\right)^3\;\frac{840\sqrt{3}\;\left(3+\eta^2\right)\;E_5\;\left\langle P_2\right\rangle - 72\left\langle P_4\right\rangle Q_4^{(3)} + 217\left\langle P_6\right\rangle Q_6^{(3)}}{266\;112\sqrt{2}} \tag{54}$$
$$\pm\,\frac{i}{24}\left(\frac{\omega_0\delta}{\omega_r}\right)^4\left[\frac{1}{2}\;\left(Q_{12}-Q_{15}\right)\left\langle P_2\right\rangle + \;\left(Q_{22}+Q_{210}\right)\left\langle P_4\right\rangle - \frac{1}{2}\,Q_3\left\langle P_6\right\rangle - \frac{119}{143}\,Q_4\left\langle P_8\right\rangle\right]$$

$$S_{\pm 2;4} = \pm i\;\left(\frac{\omega_0\delta}{\omega_r}\right)^2\frac{1}{576}\;{Q_4}^{(2)}\left\langle P_4\right\rangle \pm \frac{i}{24}\;\left(\frac{\omega_0\delta}{\omega_r}\right)^4\left[\left(Q_{211}-\frac{1}{8}Q_{28}\right)\left\langle P_4\right\rangle + \frac{523}{120}\,Q_3\left\langle P_6\right\rangle + \frac{1\;157}{1\;040}\,Q_4\left\langle P_8\right\rangle\right] \tag{55}$$

$$S_{\pm 2;6} = -\;i\;\left(\frac{\omega_0\delta}{\omega_r}\right)^3\frac{\left\langle P_6\right\rangle Q_6^{(3)}}{4608\sqrt{2}} \pm i\;\left(\frac{\omega_0\delta}{\omega_r}\right)^4\left[\frac{11}{1\;440}\,Q_3\left\langle P_6\right\rangle + \frac{1}{240}\,Q_4\left\langle P_8\right\rangle\right] \tag{56}$$

$$S_{\pm 2;8} = \mp\,\frac{i}{768}\;\left(\frac{\omega_0\delta}{\omega_r}\right)^4 Q_4\left\langle P_8\right\rangle \tag{57}$$

D. Belonging to $I_{\pm 3}$

$$C_{\pm 30} = I_3^{(\mathrm{iso})} - \left(\frac{\omega_0\delta}{\omega_r}\right)^4\left(\frac{1}{264}\,Q_{11}\left\langle P_2\right\rangle + \frac{1}{24}Q_{212}\left\langle P_4\right\rangle - \frac{17}{1\;512}\,Q_3\left\langle P_6\right\rangle + \frac{14}{10\;296}\,Q_4\left\langle P_8\right\rangle\right) \tag{58}$$

$$C_{\pm 3;2} = \pm\left(\frac{\omega_0\delta}{\omega_r}\right)^3\frac{44\sqrt{3}\left(3+\eta^2\right)\left\langle P_2\right\rangle\;E_5\;-5\left\langle P_4\right\rangle Q_4^{(3)} + 14\left\langle P_6\right\rangle Q_6^{(3)}}{33\;264\sqrt{2}}$$
$$+\left(\frac{\omega_0\delta}{\omega_r}\right)^4\left[\frac{1}{24}Q_{14}\left\langle P_2\right\rangle - \frac{1}{24}Q_{26}\left\langle P_4\right\rangle - \frac{1}{48}\,Q_3\left\langle P_6\right\rangle + \frac{41}{3\;432}\,Q_4\left\langle P_8\right\rangle\right] \tag{59}$$

$$C_{\pm 3;4} = \pm\left(\frac{\omega_0\delta}{\omega_r}\right)^3\frac{\left\langle P_4\right\rangle Q_4^{(3)} + 6\left\langle P_6\right\rangle Q_6^{(3)}}{4\;752\sqrt{2}} - \left(\frac{\omega_0\delta}{\omega_r}\right)^4\left[\frac{1}{24}Q_{27}\left\langle P_4\right\rangle + \frac{73}{720}\,Q_3\left\langle P_6\right\rangle + \frac{29}{1\;560}\,Q_4\left\langle P_8\right\rangle\right] \tag{60}$$

$$C_{\pm 3;6} = -\left(\frac{\omega_0\delta}{\omega_r}\right)^4\left[\frac{11}{360}\,Q_3\left\langle P_6\right\rangle + \frac{2}{120}\,Q_4\left\langle P_8\right\rangle\right] \tag{61}$$

$$C_{\pm 3;8} = 0 \tag{62}$$

$$S_{\pm 3;2} = \; i\;\left(\frac{\omega_0\delta}{\omega_r}\right)^3\frac{44\sqrt{3}\left(3+\eta^2\right)\left\langle P_2\right\rangle\;E_5\;-5\left\langle P_4\right\rangle Q_4^{(3)} + 14\left\langle P_6\right\rangle Q_6^{(3)}}{33\;264\sqrt{2}}$$
$$\pm\,i\;\left(\frac{\omega_0\delta}{\omega_r}\right)^4\left[\frac{1}{24}Q_{14}\left\langle P_2\right\rangle - \frac{1}{24}Q_{26}\left\langle P_4\right\rangle - \frac{1}{48}\,Q_3\left\langle P_6\right\rangle + \frac{41}{3\;432}\,Q_4\left\langle P_8\right\rangle\right] \tag{63}$$

$$S_{\pm 3;4} = i\;\left(\frac{\omega_0\delta}{\omega_r}\right)^3\frac{\left\langle P_4\right\rangle Q_4^{(3)} + 6\left\langle P_6\right\rangle Q_6^{(3)}}{4\;752\sqrt{2}} \mp i\;\left(\frac{\omega_0\delta}{\omega_r}\right)^4\left[\frac{1}{24}Q_{27}\left\langle P_4\right\rangle + \frac{73}{720}\,Q_3\left\langle P_6\right\rangle + \frac{29}{1\;560}\,Q_4\left\langle P_8\right\rangle\right] \tag{64}$$

$$S_{\pm 3;6} = \mp\,i\;\left(\frac{\omega_0\delta}{\omega_r}\right)^4\left[\frac{11}{360}\,Q_3\left\langle P_6\right\rangle + \frac{2}{120}\,Q_4\left\langle P_8\right\rangle\right] \tag{65}$$

$$S_{\pm 3;8} = 0 \tag{66}$$

The Q_α^β are geometry factors, which similar to $E_1...E_5$ contain information about the orientation of the CST PAF with respect to the segment vector. Their definitions are given in the following, where $P_n(x)$ are the Legendre polynomials and $P_n^{(m)}$ are the assigned Legendre polynomials:

$$\begin{aligned} Q_2^{(2)} &= \frac{3-\eta^2}{7} P_2(\cos\alpha) - \frac{\eta}{7} P_2^{(2)}(\cos\alpha)\ \cos 2\psi \\ Q_4^{(2)} &= -\frac{18+\eta^2}{70} P_4(\cos\alpha) - \frac{\eta}{70} P_4^{(2)}(\cos\alpha)\ \cos 2\psi - \frac{\eta^2}{48\ \cdot\ 35} P_4^{(4)}(\cos\alpha)\ \cos 4\psi \end{aligned} \tag{67}$$

$$Q_4^{(3)} = 3\sqrt{2}\ \left(9 - 4\,\eta^2\right)\ P_4(\cos\alpha) - \frac{\sqrt{2}}{4}\eta\ \left(3+\eta^2\right)\ P_4^{(2)}(\cos\alpha)\ \cos 2\psi - \frac{\sqrt{2}}{8}\eta^2\ P_4^{(4)}(\cos\alpha)\ \cos 4\psi \tag{68}$$

$$\begin{aligned} Q_6^{(3)} &= -\frac{3\sqrt{2}}{4}\ \left(6+\eta^2\right)\ P_6(\cos\alpha) - \frac{\sqrt{2}}{240}\eta\ \left(36+\eta^2\right)\ P_6^{(2)}(\cos\alpha)\ \cos 2\psi - \frac{\sqrt{2}}{480}\eta^2\ P_6^{(4)}(\cos\alpha)\ \cos 4\psi \\ &\quad - \frac{\sqrt{2}}{17\ 280}\eta^3\ P_6^{(6)}(\cos\alpha)\ \cos 6\psi \end{aligned} \tag{69}$$

$$Q_{11} = \frac{1}{1134}\left[\left(-27 - 9\eta^2 + 4\eta^4\right) P_2(\cos\alpha) + \frac{1}{2}\eta\left(27 + 5\eta^2\right) P_2^{(2)}(\cos\alpha)\cos 2\psi\right] \tag{70}$$

$$Q_{12} = \frac{1}{1386}\left[\left(315 + 72\,\eta^2 - 43\,\eta^4\right) P_2(\cos\alpha) + \eta\left(141 + 31\,\eta^2\right) P_2^{(2)}(\cos\alpha)\cos 2\psi\right] \tag{71}$$

$$Q_{13} = \frac{1}{2079}\left[\left(-405 - 36\eta^2 + 49\eta^4\right) P_2(\cos\alpha) + \eta\left(153 + 43\eta^2\right) P_2^{(2)}(\cos\alpha)\cos 2\psi\right] \tag{72}$$

$$Q_{14} = \frac{1}{1386}\left[\left(9 + 36\eta^2 - 5\eta^4\right) P_2(\cos\alpha) + \eta\left(-21 + \eta^2\right) P_2^{(2)}(\cos\alpha)\cos 2\psi\right] \tag{73}$$

$$Q_{15} = \frac{1}{8316}\left[\left(81 - 72\,\eta^2 - \ \eta^4\right) P_2(\cos\alpha) + \eta\left(9 - 13\,\eta^2\right) P_2^{(2)}(\cos\alpha)\cos 2\psi\right] \tag{74}$$

$$\begin{aligned} Q_{21} &= \frac{1}{960\ 960}\Big[\left(213\ 678 + 111\ 357\eta^2 + 817\eta^4\right) P_4(\cos\alpha) + \eta\left(17\ 523 + 3\ 329\,\eta^2\right) P_4^{(2)}(\cos\alpha)\cos 2\psi \\ &\quad + \frac{\eta^2}{24}\left(6\ 219 + 4\ 585\eta^2\right) P_4^{(4)}(\cos\alpha)\cos 4\psi\Big] \end{aligned} \tag{75}$$

$$\begin{aligned} Q_{22} &= \frac{1}{2\ 772}\Big[\left(756 + 249\,\eta^2 + 19\,\eta^4\right) P_4(\cos\alpha) + 3\,\eta\ \left(11 + 5\,\eta^2\right) P_4^{(2)}(\cos\alpha)\cos 2\psi \\ &\quad + \frac{\eta^2}{24}\left(51 + 13\,\eta^2\right) P_4^{(4)}(\cos\alpha)\cos 4\psi\Big] \end{aligned} \tag{76}$$

$$\begin{aligned} Q_{23} &= \frac{1}{123\ 552}\Big[\left(-2\ 754 - 3\ 375\eta^2 + 205\eta^4\right) P_4(\cos\alpha) + \frac{\eta}{5}\ \left(-3\ 069 + \eta^2\right) P_4^{(2)}(\cos\alpha)\cos 2\psi \\ &\quad + \frac{\eta^2}{120}\left(1\ 539 - 511\,\eta^2\right) P_4^{(4)}(\cos\alpha)\cos 4\psi\Big] \end{aligned} \tag{77}$$

$$Q_{24} = \frac{1}{720\ 720} \Big[\left(100\ 278 + 57\ 177\ \eta^2 - 163\ \eta^4\right)\ P_4\left(\cos\alpha\right) + \eta\ \left(9207 + 1\ 453\ \eta^2\right)\ P_4^{(2)}\left(\cos\alpha\right)\cos 2\psi$$
$$+ \frac{\eta^2}{24}\left(1\ 935\ +\ 2\ 261\eta^2\right)\ P_4^{(4)}\left(\cos\alpha\right)\cos 4\psi \Big] \tag{78}$$

$$Q_{25} = \frac{1}{54\ 054} \Big[\left(11\ 664 + 3\ 771\ \eta^2 + 301\ \eta^4\right)\ P_4\left(\cos\alpha\right) + \eta\ \left(495 + 233\ \eta^2\right)\ P_4^{(2)}\left(\cos\alpha\right)\cos 2\psi$$
$$+ \frac{\eta^2}{24}\left(801\ +\ 199\ \eta^2\right)\ P_4^{(4)}\left(\cos\alpha\right)\cos 4\psi \Big] \tag{79}$$

$$Q_{26} = \frac{1}{36\ 036} \Big[\left(864 + 327\ \eta^2 + 17\ \eta^4\right)\ P_4\left(\cos\alpha\right) + \frac{3}{5}\eta\ \left(77 + 27\ \eta^2\right)\ P_4^{(2)}\left(\cos\alpha\right)\cos 2\psi$$
$$+ \frac{\eta^2}{120}\left(249\ +\ 79\ \eta^2\right)\ P_4^{(4)}\left(\cos\alpha\right)\cos 4\psi \Big] \tag{80}$$

$$Q_{27} = \frac{1}{123\ 552} \Big[\left(3402 - 2493\ \eta^2 + 487\ \eta^4\right)\ P_4\left(\cos\alpha\right) + \frac{1}{5}\eta\ \left(-2871 + 739\ \eta^2\right)\ P_4^{(2)}\left(\cos\alpha\right)\cos 2\psi$$
$$+ \frac{\eta^2}{120}\left(4761\ -\ 109\ \eta^2\right)\ P_4^{(4)}\left(\cos\alpha\right)\cos 4\psi \Big] \tag{81}$$

$$Q_{28} = \frac{1}{123\ 552} \Big[\left(810 + 387\ \eta^2 + 7\ \eta^4\right)\ P_4\left(\cos\alpha\right) + \frac{1}{5}\eta\ \left(297 + 67\ \eta^2\right)\ P_4^{(2)}\left(\cos\alpha\right)\cos 2\psi$$
$$- \frac{\eta^2}{120}\left(153\ +\ 83\ \eta^2\right)\ P_4^{(4)}\left(\cos\alpha\right)\cos 4\psi \Big] \tag{82}$$

$$Q_{29} = \frac{1}{1\ 441\ 440} \Big[\left(-31914 - 32\ 751\ \eta^2 + 1\ 669\ \eta^4\right)\ P_4\left(\cos\alpha\right) - \eta\ \left(5\ 841 + 139\ \eta^2\right)\ P_4^{(2)}\left(\cos\alpha\right)\cos 2\psi$$
$$+ \frac{\eta^2}{24}\left(2\ 295\ -\ 1\ 043\ \eta^2\right)\ P_4^{(4)}\left(\cos\alpha\right)\cos 4\psi \Big] \tag{83}$$

$$Q_{210} = \frac{1}{216\ 216} \Big[\left(972 + 207\ \eta^2 + 37\ \eta^4\right)\ P_4\left(\cos\alpha\right) + \frac{\eta}{5}\ \left(99 + 109\ \eta^2\right)\ P_4^{(2)}\left(\cos\alpha\right)\cos 2\psi$$
$$+ \frac{\eta^2}{120}\left(441\ +\ \ 71\ \eta^2\right)\ P_4^{(4)}\left(\cos\alpha\right)\cos 4\psi \Big] \tag{84}$$

$$Q_{211} = \frac{1}{494\ 208} \Big[\left(30\ 942 - 9\ 927\ \eta^2 + 3\ 013\ \eta^4\right)\ P_4\left(\cos\alpha\right) + \eta\ \left(-2673 + 1061\ \eta^2\right)\ P_4^{(2)}\left(\cos\alpha\right)\cos 2\psi$$
$$+ \frac{\eta^2}{24}\left(6111\ +\ \ 85\ \eta^2\right)\ P_4^{(4)}\left(\cos\alpha\right)\cos 4\psi \Big] \tag{85}$$

$$Q_{212} = \frac{1}{240\ 240} \Big[\left(1\ 458 - 333\ \eta^2 + 127\ \eta^4\right)\ P_4\left(\cos\alpha\right) + \eta\ \left(-99 + 47\ \eta^2\right)\ P_4^{(2)}\left(\cos\alpha\right)\cos 2\psi$$
$$+ \frac{\eta^2}{24}\left(261\ +7\ \eta^2\right)\ P_4^{(4)}\left(\cos\alpha\right)\cos 4\psi \Big] \tag{86}$$

$$Q_3 = \frac{1}{3\ 168}\left[\left(-54+27\eta^2+\eta^4\right)P_6\left(\cos\alpha\right)+\frac{\eta^3}{2}P_6^{(2)}\left(\cos\alpha\right)\cos 2\psi+\frac{\eta^2\ \left(9+\eta^2\right)}{360}P_6^{(4)}\left(\cos\alpha\right)\cos 4\psi\right.$$
$$\left.+\frac{\eta^3}{720}P_6^{(6)}\left(\cos\alpha\right)\cos 4\psi\right] \tag{87}$$

$$Q_4 = \frac{1}{6\ 912}\left[\left(216+72\ \eta^2+\eta^4\right)P_8\left(\cos\alpha\right)+\frac{3}{7}\eta\left(12+\eta^2\right)P_8^{(2)}\left(\cos\alpha\right)\cos 2\psi\right.$$
$$\left.+\frac{\eta^2\ \left(54+\eta^2\right)}{1\ 260}P_8^{(4)}\left(\cos\alpha\right)\cos 4\psi+\frac{\eta^3}{2\ 520}P_8^{(6)}\left(\cos\alpha\right)\cos 4\psi+\frac{\eta^4}{120\ 960}P_8^{(8)}\left(\cos\alpha\right)\cos 4\psi\right] \tag{88}$$

S6. OSCILLATION COEFFICIENTS: EXPERIMENTAL DATA

Table I. C7 = CH_3; all values ±0.0016

m	C_{m0}	C_{m2}	S_{m2}
-2		- 0.00485	0.0058
-1	0.0133	0	0
0	0.9700	0.003395	- 0.0107
1	0.0126	0	0
2		0.003395	0.0061

Table II. C1+C2+C5 = CO_3 and the two C_q; all values ±0.001

m	C_{m0}	C_{m2}	S_{m2}
-3	0.014	0	0.0024
-2	-	-0.0094	0.0087
-1	0.160	-0.0113	0.0078
0	0.597	0.0081	-0.0263
1	0.167	-0.0070	-0.0130
2	0.0375	0.0184	0.0167
3	0.0045	0.0031	0.0032

Table III. C3 = CH; all values ±0.0011

m	C_{m0}	C_{m2}	S_{m2}
-3	0.0048	0	0
-2	0.0416	-0.0067	0.0067
-1	0.1153	0.0080	-0.0089
0	0.6369	-0.0061	-0.0051
1	0.1834	0.0080	0.0085
2	0.0156	0	0
3	0.0024	0	0

Table IV. C4 = CH; all values ±0.0011

m	C_{m0}	C_{m2}	S_{m2}
-3	0.0083	0	0
-2	0.0607	-0.0094	0.0089
-1	0.1508	0.0127	-0.0132
0	0.6675	-0.0089	-0.0077
1	0.0868	0.0117	0.0125
2	0.0230	0	0
3	0.0028	0	0
